# Supplementary material for: Retrograde signaling by a mtDNA-encoded non-coding RNA preserves mitochondrial bioenergetics
Source: Commun Biol. 2020 Oct 30;3:626. doi: 10.1038/s42003-020-01322-4 (PMC7603330; doi:10.1038/s42003-020-01322-4)
Supplement: Supplementary file 1 — Supplementary Information [file 42003_2020_1322_MOESM1_ESM.pdf]

Supplementary Figures

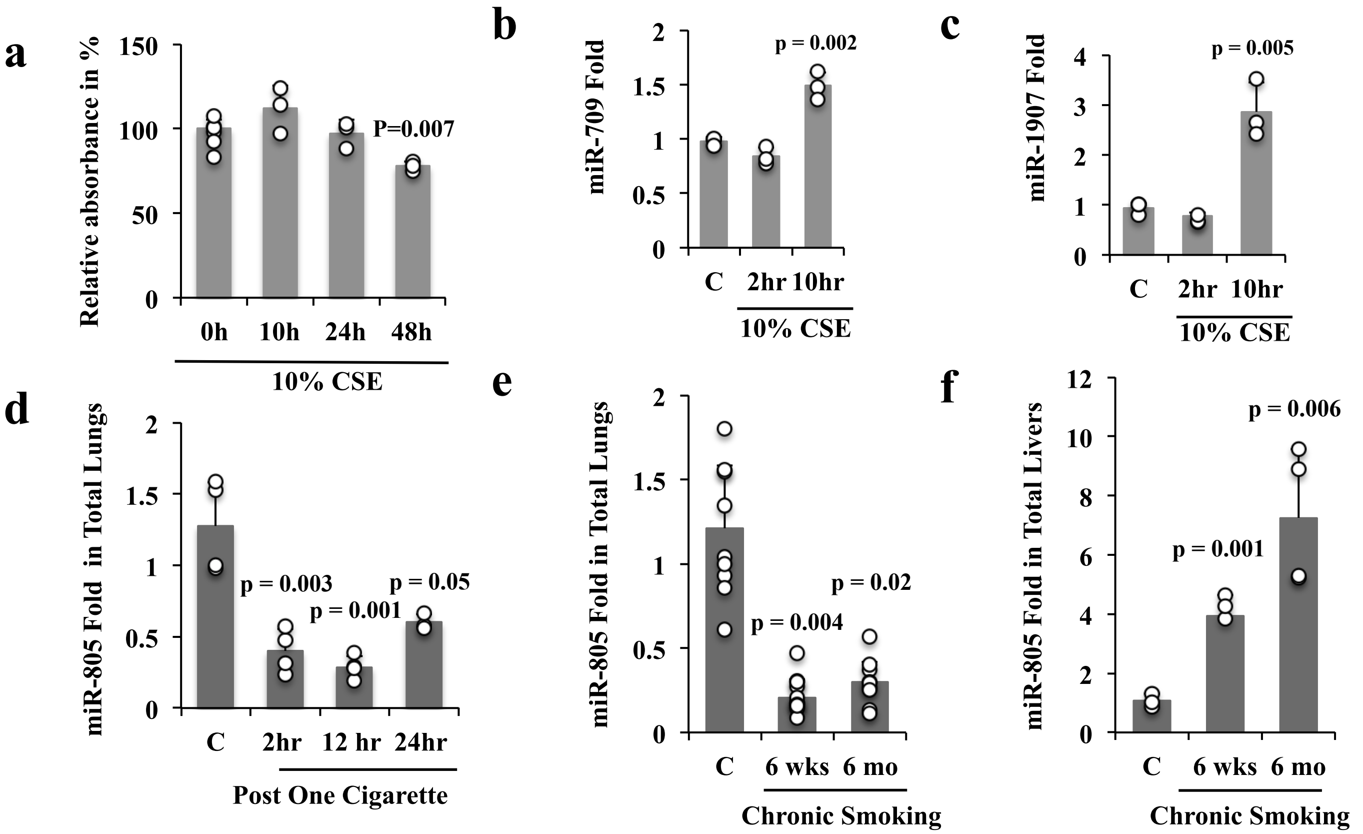

Supplemental Figure 1. ***(a-c) Cell viability and validation of miRNAs identified by the microarray screen.*** **(a)** Cell viability after exposure to 10% CSE for 10, 24, and 48 hr was tested using the Titer-Glo assay, in at least 3 independent experiments **(b-c)** Cultured MLE12 cells were exposed or not to 10% CSE for 2 hr and 10hr and levels of **(b)** miR-709 (Control n=3, 2hr n=3, and 10hr n=3), and **(c)** miR-1907 **(**Control n=3, 2hr n=3**;** 10hr n=3) were tested by RT-qPCR. Please note, miR-1907 has been reported to be induced during liver damage, and contributes to liver regeneration by stimulating productive autophagy[1]. In addition, miR-1907 and miR-709 have been reported to participate in the regulation of apoptosis[2,3]. **(d-f*****) miR-805 up-regulation in response to CS-exposure is cell type dependent.*** miRNAs were extracted and miR805 expression levels were analyzed by RT-qPCR in **(d)** Total lung lysates obtained from C57BL/6 female mice exposed or not to 1 cigarette, and then sacrificed after 2 hr (n=4 animal), 12 hours (n=4, animals), and 24 hr (n=3 animals) post-exposure. **(e)** Total lung lysates obtained from C57BL/6 mice chronically exposed or not to CS for 6 weeks (Control n=3 animals, 6 weeks CS-exposed mice n=4) and 6 months (Control n=3, 6 months CS-exposed mice n=4) and sacrificed 16 hr after last exposure. **(f)** Total liver lysates obtained from C57BL/6 mice chronically exposed or not to CS for 6 weeks (Control-n=3 animals, 6 weeks CS-exposed mice n=4 animals), and for 6 months (n=4 animals) and sacrificed 16 hr after last exposure. RT-qPCR levels of miRs were normalized to sno55RNA in all panels, folds calculated to respective controls.

a **b** **c** **d**

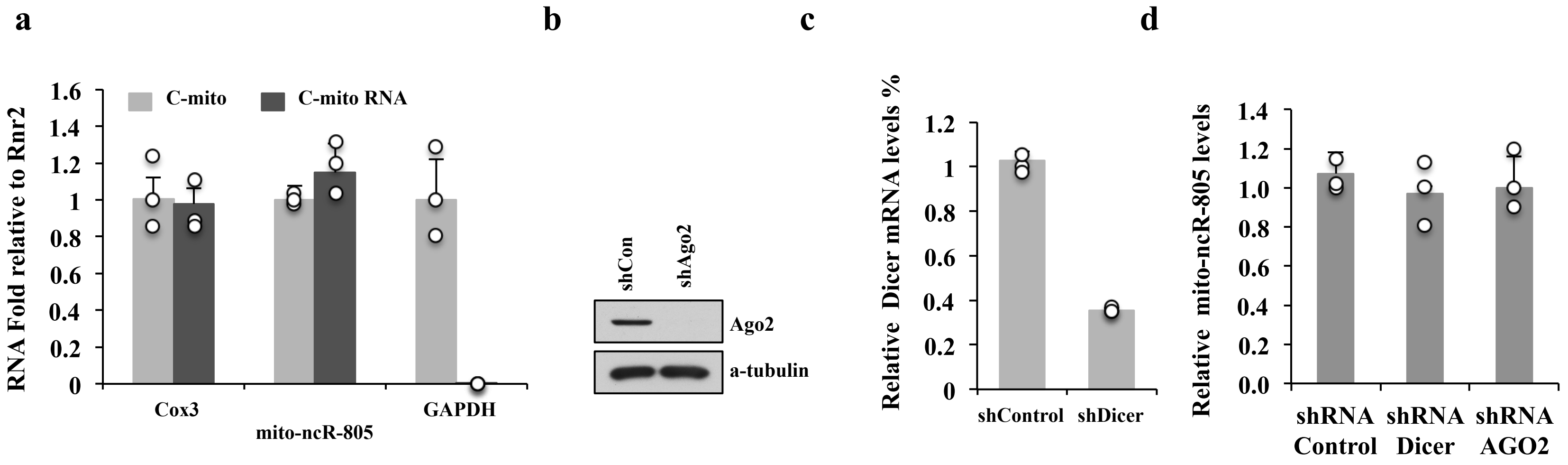

e **f**

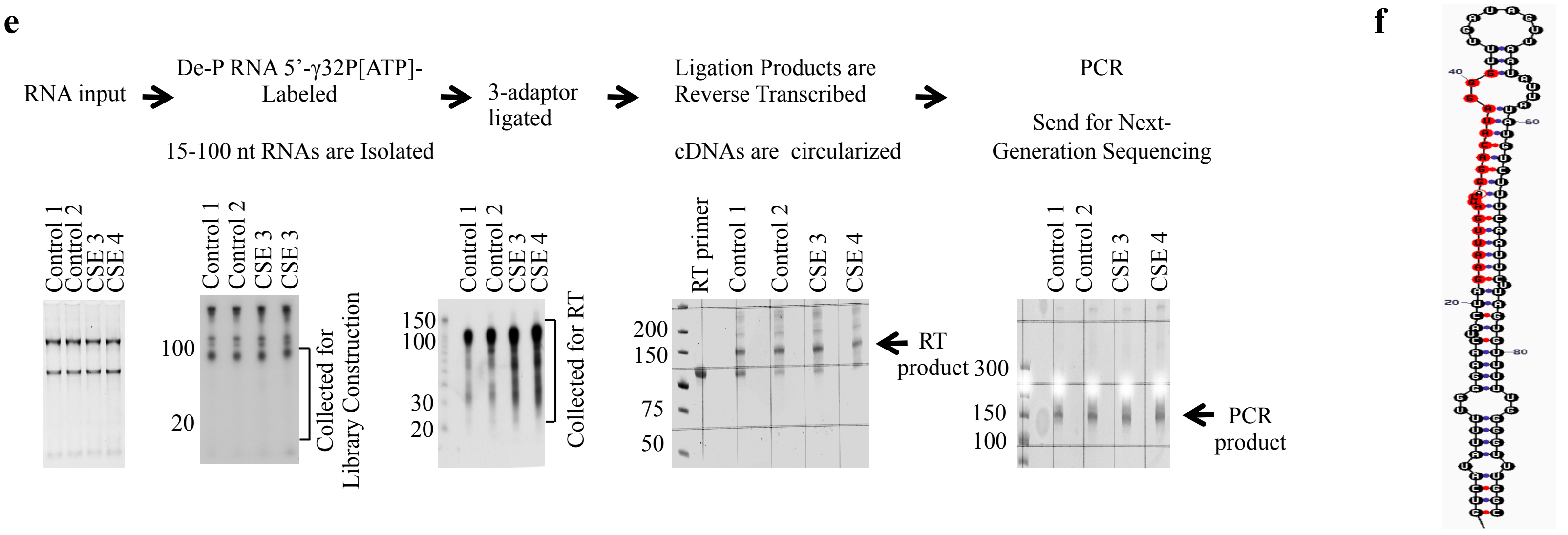

g

Sample	chrM: 16115-16209	Mm10 Genome
C1 (GCT)	1233	4
C2 (TAG)	857	3
CSE1 (CGG)	4658	9
CSE2 (AAT)	3965	11

h

Gene name	Homology
NM_001166648.2: ZFP280c	1739 - CAAGTTCTTAGTGTTTT - 1723
mmu-mitos-ncR-805	16188 - GAATTGATCAGGACATAGGGTTTGATAGTTAATATTATATGTCTTTCAAGTTCTTAGTGTTTTTGGGGTT - 16116
	||||||||||||* |||* ||||
NM_175314.4: Adamts9	7267 - TAATATTATATGTTTTTAAAGT - 7288

i

Organism	Length of mtDNA	D-loop Defined	D-loop Coordinates	Sequence
H. sapiens	16,569 kb	Yes	1-576, 16024-16569	16516 - GAAGTAGGAACCAGATGTCG - 16497; 369 - GTTCTT - - TGTTTTTGGGGTT - 350; 2996 - GATCAGGACAT - 3006; 568 - GTCTGTTTTGGGGTT - 555; 11323 - GTTTGATAGTT - 11313
M. musculus	16,300 kb	Yes	15424-16300	**16188** - GAATTGATCAGGACATAGGGTTTGATAGTTAATATTATATGTCTTTCAAGTTCTTAGTGTTTTTGGGGTT - **16116**
Danio Rerio (Zebrafish)	16,596 kb	Yes	1-950	3483 - GATCAGGACAT - 3493; 14442 - TAGTGTTTTTG - 14432
D. melanogaster	14,913 kb	No		4187 - TTAATATTATATGTCTTTCAAGTT- 4208; 9980 - TTAATATTATAT- 9991; 13777 - TAATATTATAT- 13767
C. elegans	12,976 kb	No		2904 - GTTT - A - AGTTAATATTA- 2921; 6628 -TAATATTATATATGTTT- 6644; 10621 -TTCAAGTTCTT- 10611
S. pombe	19,431 kb	No		102 – AGTTAATATTA - 112; 18975 – TTTGATAGTTA - 18985
S. cerevisiae	85,779 kb	No		– TTTAATTATTAATATTATATTTTTTT – 3x through-out mtDNA; –TAATTATTATATT- more than 50x through-out mtDNA

j

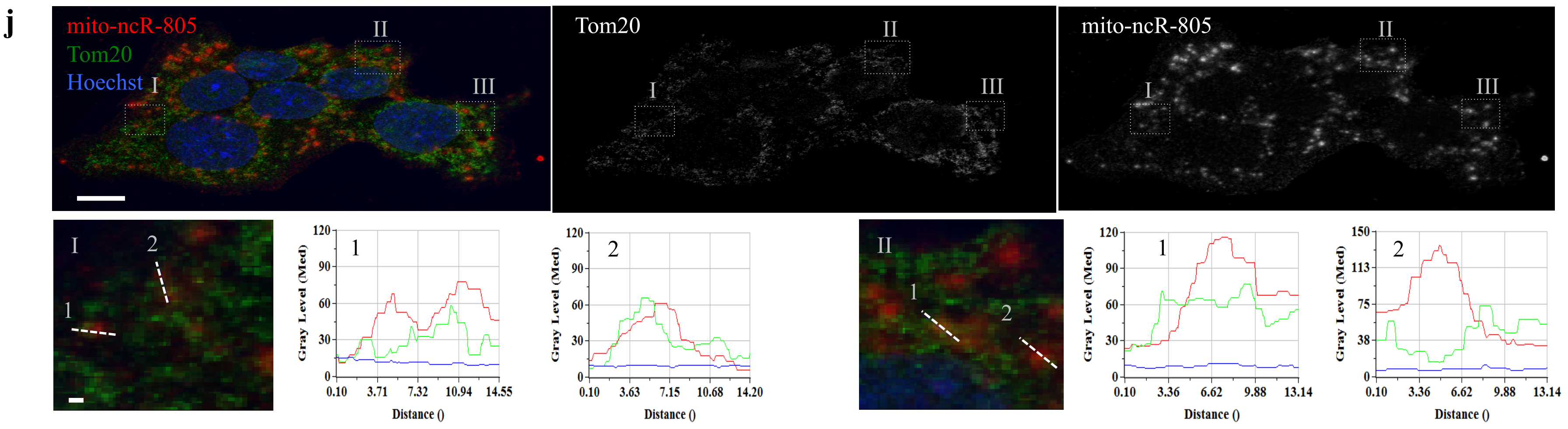

Supplemental Figure 2. ***Ago2 and Dicer depletion, library construction for RNAseq and predicted miR-805 secondary structure.*** **(a)** Mitochondrial and cytosolic fractions were isolated from MLE12 cells, and treated or not with RNase as indicated. Levels of mito-ncR-805, Cox3, and GAPDH were normalized to the levels of mitochondria encoded 16S rRNA, Rnr2. Folds were calculated to the respective RNase untreated fractions. **(b-c)** MLE12 cells after two rounds of lentiviral infection with viruses carrying shRNA of non-targeting Control, Ago2 and Dicer sequences. After selection, proteins or RNA was extracted from established cultures, and levels of Ago2 were tested by Western blot analysis (b), and levels of Dicer mRNA were tested by RT-qPCR (c). **(d)** mito-ncR-805 expression levels in shRNA Control, shRNA AGO2, and shRNA Dicer. **(e)** Schematic presentation of RNA library construction. Quality of input RNA was analyzed using a bio-analyzer and found between 9.5 and 10. The final library quality was tested again on the bio-analyzer, and no degradation products were detected. **(f)** Predicted Stem-loop structure of pre-miR-805 was created using a mFold RNA folding program at miRNAMap[4] http://mirnamap.mbc.nctu.edu.tw/php/mirna_entry.php?acc=MI0005204 . **(g)** Alignment of all of the sequence reads obtained from RNA library against the entire mouse nuclear and mitochondrial genomes using BLASTN. **(h)** Nuclear homology of mito-ncR-805. **(i)** Evolutional conservation of mito-ncR-805 **(j).** MLE12 cells were grown on slides, fixed, hybridized with the mito-ncR-805 specific probe, and followed by Tom20 staining. Confocal images were acquired using 63x magnification and HuVolution function of the Leica SP8-HyVolution laser scanning confocal microscope. A representative image, which is a projection of 14 consecutive planes acquired at the distance of 0.13 μm. Scale bar is 10 μm; boxed regions I-II are shown enlarged on the bottom panel, as a single plane chosen for its maximal signal intensity for each channel, accompanied by linescanes and plots of three channel intensities through regions depicted. Scale bar is 1 μm.

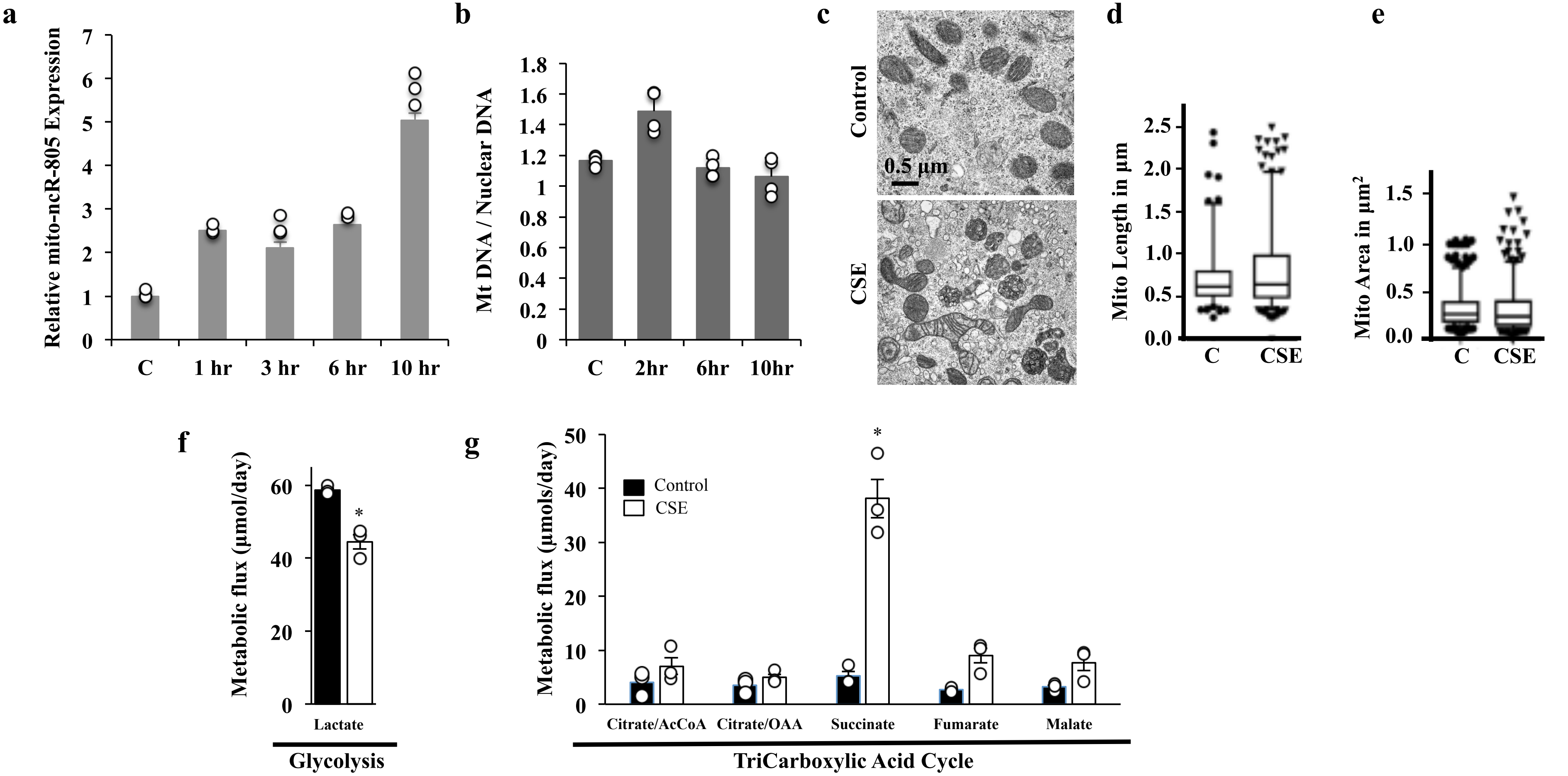

Supplemental Figure 3. ***Induction of mito-ncR-805 and mitochondrial morphology in response to 6% CSE-treatment.*** **(a)** MLE 12 cells were seeded in 35 mm in diameter plates, containing 2 18mm covers slips. Cells were exposed or not to 6% CSE for the indicated times. Slides were removed and fixed for FISH analysis (Fig. 4); RNA from the remaining cells was extracted, and analyzed for the expression levels of mito-ncR-805 by RT-qPCR. **(b)** MLE12 cells were exposed or not to CSE, DNA was extracted and mitochondrial copy number was determined (n=3 independent experiments, each of two repetitions, for each time point, $p^{2hr/C}$=0.0006, $p^{10hr/C}$ =0.03, $p^{10hr/C}$ =0.5. **(c)** TEM analysis of mitochondria in MLE12 cells exposed or not to CSE for 6h**. (d-e)** Quantitation of (c). Mitochondria length (d) and area (e) were measured in 200 mitochondria from 3 different cells using ImageJ. $p(C/CSE)^{length}$=3,0407E-221, $p(C/CSE)^{area}$=3,0809E-143.**(f-g)** MLE12 cells were exposed or not to 6% CSE in the media containing to 20 mM [U-^{13}C]glucose for 6 hours, harvested and metabolic flux was determined following ^{13}C label incorporation into various metabolites. Metabolic flux of glycolysis (f), TCA (g). p-values for n=3 independent experiments are $C/CSE^{lactate}$ = 0.06, and $C/CSE^{succinate}$ = 0.018.

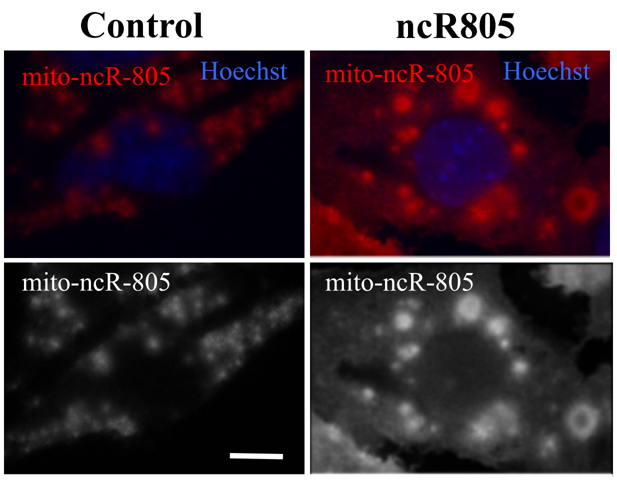

Supplemental Figure 4. ***Over-expression of full-length (ncR805) mito-ncR-805.*** Cells were transfected with ncR805 or with non-targeting RNA fixed and processed for FISH detection of ncR805. Please note that in transfected with ncR805 cells nuclear localization of ncR805 can be detected. Scale bar 10 uM. We also attempted to design two additional 20-bp inhibitors that target other than AI805 regions of mito-ncR-805, but those were less specific, required an order of magnitude higher concentration to reach a lesser inhibition, and affected cell survival**.** Therefore their biological effects were not investigated.

Fig. 1a blots

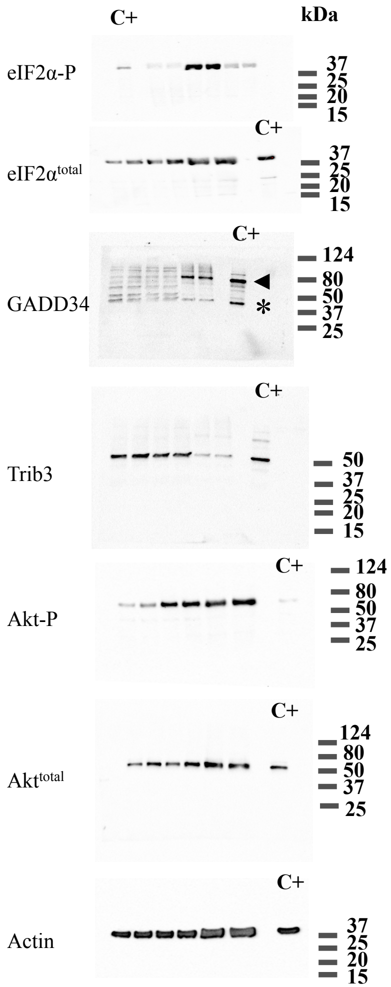

Fig. 2b blots

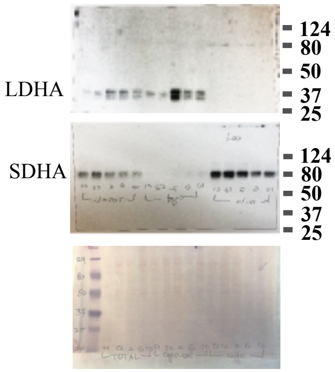

Fig. 2e blots

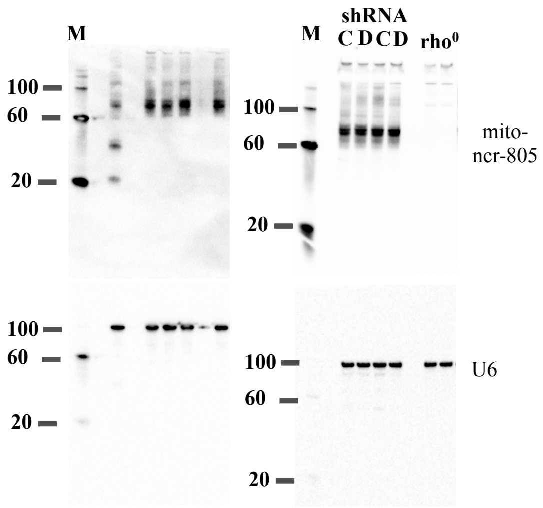

Fig. 4e blots

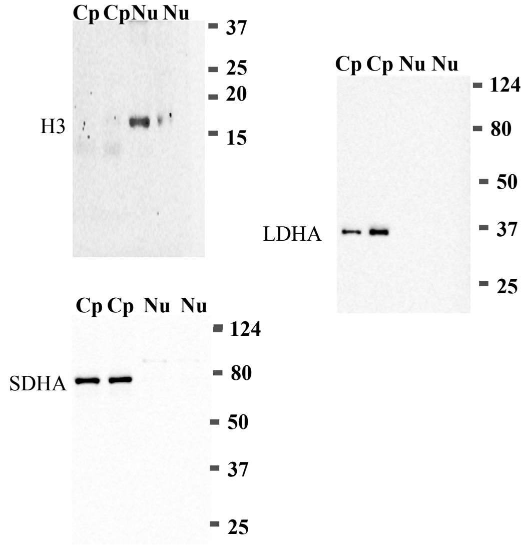

Fig. 4f and 6a blots

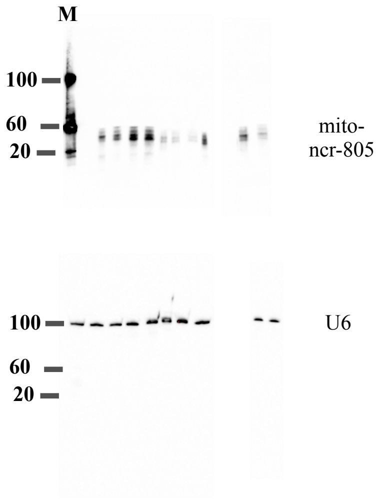

Fig. 6f blots

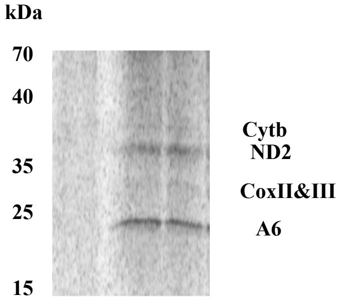

Suppl. Fig. 2b blots

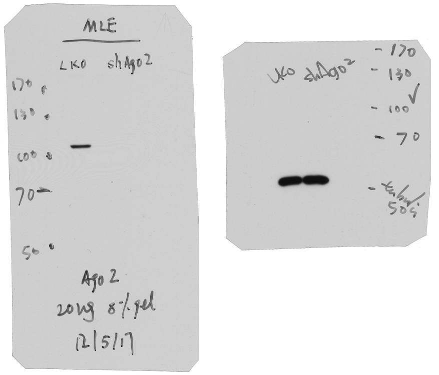

Supplemental Figure 5. Original blots for all Western and Northern blots performed during the course of this stud y. * in the blot of GADD34 for Fig. 1a indicates non-specific band, arrow indicates GADD34 specific band.

References

1 Lu, T. *et al.* Partial Hepatectomy-Induced Upregulation of miR-1907 Accelerates Liver Regeneration by Activation Autophagy. *Biomed Res Int* **2018**, 3817057, doi:10.1155/2018/3817057 (2018).

2 Tang, R. *et al.* Mouse miRNA-709 directly regulates miRNA-15a/16-1 biogenesis at the posttranscriptional level in the nucleus: evidence for a microRNA hierarchy system. *Cell Res* **22**, 504-515, doi:10.1038/cr.2011.137 (2012).

3 Zhao, J., Ou, S. L., Wang, W. Y., Yan, C. & Chi, L. X. MicroRNA-1907 enhances atherosclerosis-associated endothelial cell apoptosis by suppressing Bcl-2. *Am J Transl Res* **9**, 3433-3442 (2017).

4 Zuker, M. Mfold web server for nucleic acid folding and hybridization prediction. *Nucleic Acids Res* **31**, 3406-3415 (2003).
