# Supplementary material for: Retrograde signaling by a mtDNA-encoded non-coding RNA preserves mitochondrial bioenergetics
Source: Commun Biol. 2020 Oct 30;3:626. doi: 10.1038/s42003-020-01322-4 (PMC7603330; doi:10.1038/s42003-020-01322-4)
Supplement: Supplementary file 2 — Description of Additional Supplementary Files [file 42003_2020_1322_MOESM2_ESM.pdf]

Description of Additional Supplementary Files

File Name: Supplementary Data 1

Description:

Sup. Sheet 1: Excel table of changes in fold expression for all 627 miRs screened

Sup. Sheet 2: all qPCR and RNA-seq data

Sup. Sheet 3: Data from all genes tested by Mitochondrial Energy Metabolism RT2 Profiler PCR Arrays.
Graphs: Scatter plot comparison of normalized to GAPDH gene expression in control and AI805 transfected MLE12 cells obtained using Mitochondrial Energy Metabolism RT2 Profiler PCR Arrays. The central line indicates unchanged gene expression. The dotted lines indicate the 2-fold regulation threshold. Data points below the dotted lines in the lower right section (blue) are the genes expression of which changed >= 2 folds.

Sup. Sheet 4: Data for Fig. 4d, 5e, 6g, 7f. Counting of mito-ncR-805 dots in MLE12 cells, and in AETII and non-AETII cells in vivo. Mitochondria length and area measurements.

Sup. Sheet 5: Data on morphometry measurements

Sup. Sheet 6-8: Metabolomics data for the main figure 7.
Page 1 - raw data on AI805 and calculation of metabolites concentrations
Page 2 - calculation of absolute rates of metabolites
Page 3 raw data on ncR805 data

Sup. Sheet 9: Mitochondrial bioenergetics measurements and calculations

Sup. Sheet 10: Metabolomics data for Supplementary figure 3.
