# Supplementary material for: Retrograde signaling by a mtDNA-encoded non-coding RNA preserves mitochondrial bioenergetics
Source: Commun Biol. 2020 Oct 30;3:626. doi: 10.1038/s42003-020-01322-4 (PMC7603330; doi:10.1038/s42003-020-01322-4)
Supplement: Supplementary file 5 — Reporting Summary [file 42003_2020_1322_MOESM5_ESM.pdf]

nature research

Corresponding author(s): Anna Blumental-Perry, Maria Hatzoglou

Last updated by author(s): Aug 11, 2020

Reporting Summary

Nature Research wishes to improve the reproducibility of the work that we publish. This form provides structure for consistency and transparency in reporting. For further information on Nature Research policies, see Authors & Referees and the Editorial Policy Checklist.

Statistics

For all statistical analyses, confirm that the following items are present in the figure legend, table legend, main text, or Methods section.

n/a	Confirmed	
☐	☒	The exact sample size (*n*) for each experimental group/condition, given as a discrete number and unit of measurement
☐	☒	A statement on whether measurements were taken from distinct samples or whether the same sample was measured repeatedly
☐	☒	The statistical test(s) used AND whether they are one- or two-sided<br>*Only common tests should be described solely by name; describe more complex techniques in the Methods section.*
☐	☒	A description of all covariates tested
☒	☐	A description of any assumptions or corrections, such as tests of normality and adjustment for multiple comparisons
☐	☒	A full description of the statistical parameters including central tendency (e.g. means) or other basic estimates (e.g. regression coefficient) AND variation (e.g. standard deviation) or associated estimates of uncertainty (e.g. confidence intervals)
☐	☒	For null hypothesis testing, the test statistic (e.g. *F*, *t*, *r*) with confidence intervals, effect sizes, degrees of freedom and *P* value noted<br>*Give P values as exact values whenever suitable.*
☒	☐	For Bayesian analysis, information on the choice of priors and Markov chain Monte Carlo settings
☐	☒	For hierarchical and complex designs, identification of the appropriate level for tests and full reporting of outcomes
☒	☐	Estimates of effect sizes (e.g. Cohen's *d*, Pearson's *r*), indicating how they were calculated

Our web collection on statistics for biologists contains articles on many of the points above.

Software and code

Policy information about availability of computer code

Data collection	Microsoft Word and Excel for Mac 14.7.7, Nikon microscope NIS Elements Software 2.34, GeneSpring GX 11.0 (Agilent Technologies), real-time PCR using Applied Biosystems 7500 software, and StepOnePlus Real-Time PCR System (Applied Biosystem), Illumina NextSeq 550 platform, Agilent 5973 mass spectrometer, Seahorse XFe96 extracellular flux analyzer (Seahorse Bioscience, USA), Biotek Synergy HT spectrophotometer.
Data analysis	Microsoft Excel, ImageJ (NIH, Bethesda, MD), miRBase 22.1, miRNAMAP 2.0, Illumina NextSeq 550 platform, BlastN (NIH, Bethesda, MD), Agilent 5973 mass spectrometer , Qiagen algorithm software for Pathway analysis available at the data analysis web portal at http://www.qiagen.com/geneglobe.

For manuscripts utilizing custom algorithms or software that are central to the research but not yet described in published literature, software must be made available to editors/reviewers. We strongly encourage code deposition in a community repository (e.g. GitHub). See the Nature Research guidelines for submitting code & software for further information.

Data

Policy information about availability of data

All manuscripts must include a data availability statement. This statement should provide the following information, where applicable:

- Accession codes, unique identifiers, or web links for publicly available datasets
- A list of figures that have associated raw data
- A description of any restrictions on data availability

The microarray dataset is available in Gene Expression Omnibus (GSE131188, https://www.ncbi.nlm.nih.gov/geo/query/acc.cgi?acc=GSE131188)
RNA-Seq dataset is available in Gene Expression Omnibus (GSE131439, https://www.ncbi.nlm.nih.gov/geo/query/acc.cgi?acc=GSE131439)

Field-specific reporting

Please select the one below that is the best fit for your research. If you are not sure, read the appropriate sections before making your selection.

- [x] Life sciences
- [ ] Behavioural & social sciences
- [ ] Ecological, evolutionary & environmental sciences

For a reference copy of the document with all sections, see nature.com/documents/nr-reporting-summary-flat.pdf

Life sciences study design

All studies must disclose on these points even when the disclosure is negative.

Sample size	Was chosen
Data exclusions	No Data was excluded
Replication	All experiments were repeated 3-6 times, number for each experiment indicated in figure legend, Data presented as mean+/-SD, p- value calculated and displayed. All experiments included positive and negative controls.
Randomization	For animals studies, groups were balanced and randomized based on age and exposure protocol.
Blinding	For animals studies investigators were blinded to group allocations.

Reporting for specific materials, systems and methods

We require information from authors about some types of materials, experimental systems and methods used in many studies. Here, indicate whether each material, system or method listed is relevant to your study. If you are not sure if a list item applies to your research, read the appropriate section before selecting a response.

Materials & experimental systems

n/a	Involved in the study	
☐	☒	Antibodies
☐	☒	Eukaryotic cell lines
☐	☐	Palaeontology
☐	☒	Animals and other organisms
☒	☐	Human research participants
☒	☐	Clinical data

Methods

n/a	Involved in the study	
☒	☐	ChIP-seq
☒	☐	Flow cytometry
☒	☐	MRI-based neuroimaging

Antibodies

Antibodies used

α-CD-16/32 (BD Biosciences, Cat. # 553143, 5 µg/isolation),
α-Ter-119 (BD Biosciences, Cat. # 553672, 7.5 µg/isolation),
α-CD45 (BD Biosciences, Cat. # 553078, 10 µg/isolation),
α -ATP-binding cassette sub-family A member 3 antibody [3C9] (Abcam, Cat. # ab24751, 1:100),
α-Ago2 (Wako Chemicals, Cat. # 018-22021, 1:500),
α-tubulin (Millipore-Sigma, Cat. # T9026, 1:5,000).
α-lactate dehydrogenase A (LDHA), (Cell Signaling, Cat # 2012, 1:1000)
α-succinate dehydrogenase subunit A (SDHA), (Abcam, Cat # ab14715, 1:1000)
α-H3 (Cell Signaling, Cat # 9715, 1:4000)
α-GADD34 (Santa Cruz, Cat. # SC-825, 1:500)
α-Actin (Abcam, Cat. # ab6276, 1:10,000)
α-Phospho-eIF2α (Ser51) (Cell Signaling, Cat. # 3597, 1:1000)
α-eIF2α total (Cell Signaling, Cat. # 9722, 1:1000)
α-Trib3 (LSBio, LifeSpan BioSciences, Cat. # LS-C164592, 1:250)
α-Phospho-Akt (Trh308), (Cell Signaling, Cat. # 2965, 1:500)
α-Akt total (Cell Signaling, Cat. # 9272, 1:1000)
α-Tom20 (Cell Signaling, Cat.# 4240b, dilution 1:40)
goat anti-mouse IgG Alexa Fluor 594 (Molecular Probes, Cat. # A11032, 1:500),
goat anti-mouse IgG Horse Radish Peroxidase (Thermo Fisher Scientific, Cat # 31430, 1:5,000),
goat anti-rabbit IgG Horse Radish Peroxidase (Thermo Fisher Scientific, Cat # 31460, 1:5,000)

Validation

All antibodies were obtained from indicated companies and tested before use for off target effects and optimal dilutions.
α-CD-16/32- BD Biosciences Website, used in 15 publication, use of biotinylated
antibody in alveolar type 2 cell isolation is well established and reviewed in Methods Mol Biol. 2018;1799:59-69. doi:

10.1007/978-1-4939-7896-0_6.
α-Ter-119 - BD Biosciences Website, used in 4 publication, use of biotinylated antibody in alveolar type 2 cell isolation is well established and reviewed in Methods Mol Biol. 2018;1799:59-69. doi: 10.1007/978-1-4939-7896-0_6.
α-CD45 - Use of biotinylated antibody in alveolar type 2 cell isolation is well established and reviewed in Methods Mol Biol. 2018;1799:59-69. doi: 10.1007/978-1-4939-7896-0_6.
α - [3C9] – Abcam Website, used in 10 publications
α-Ago2 - Wako Chemicals Website, used in 2 publications
α-tubulin Millipore-Sigma Website, used in 2035 publications
H3 antibody – Cell signaling Website, used in 461 publications
α-LDHA - Cell signaling Website, used in 49 publications
α-SDHA - Abcam Website, used in 186 publications
α-GADD34 – Santa Cruz Website, 22 citations
α-Actin - Abcam Website, used in 1285 publications
α-Phospho-eIF2α (Ser51) - Cell signaling Website, used in 185 publications
α-eIF2α total - Cell signaling Website, used in 358 publications
α-Trib3 – was validated for Western by the company, verified by us using siRNA against TRIB3.
α-Phospho-Akt (Trh308) - Cell signaling Website, used in 493 publications
α-Akt total - Cell signaling Website, used in 4406 publications
α-Tom20, Cell signaling Web site, used in 28 publication, including 5 IF

goat anti-mouse IgG Horse Radish Peroxidase - Thermo Fisher Scientific Website, used in 70 publications, 62 for Western blot
goat anti-rabbit IgG Horse Radish Peroxidase - Thermo Fisher Scientific Website, used in 37 publications, 62 for Western blot
goat anti-mouse IgG Alexa Fluor 594 – Thermo Fisher Scientific Website, used in 210 publications

Eukaryotic cell lines

Policy information about cell lines

Cell line source(s)	Mouse lung epithelial cell line 12 (MLE12), Mouse monocyte macrophage RAW 264.7 cell lines were purchased from and cultured per American Type Culture Collection (ATCC).
Authentication	MLE12 and RAW264.7 cell lines were authenticated using standard American Type Tissue Collection methods including morphology check by microscope and growth curve analysis. MLE12 cells were also tested for the presense of lamellar bodies, Raw264.7 for their ability to engulf particles.
Mycoplasma contamination	MLE12 and Raw264.7 were tested for mycoplasma contamination using the Sigma LookOut Mycoplasma PCR Detection Kit MP0035-1KT
Commonly misidentified lines (See ICLAC register)	No commonly misidentified cell lines were used.

Palaeontology

Specimen provenance	*Provide provenance information for specimens and describe permits that were obtained for the work (including the name of the issuing authority, the date of issue, and any identifying information).*
Specimen deposition	*Indicate where the specimens have been deposited to permit free access by other researchers.*
Dating methods	*If new dates are provided, describe how they were obtained (e.g. collection, storage, sample pretreatment and measurement), where they were obtained (i.e. lab name), the calibration program and the protocol for quality assurance OR state that no new dates are provided.*

☐ Tick this box to confirm that the raw and calibrated dates are available in the paper or in Supplementary Information.

Animals and other organisms

Policy information about studies involving animals; ARRIVE guidelines recommended for reporting animal research

Laboratory animals	C57BL/6 female mice from Taconic Farms were used in this study. Mice were purchased 8–10 wk old, and kept for smoke-exposure studies till 8 months of age
Wild animals	The study did not use wild animals
Field-collected samples	The study did not use samples collected in the field
Ethics oversight	Mercer University Institutional Animal Care and Use Committee approved all animals protocols

Note that full information on the approval of the study protocol must also be provided in the manuscript.
